# The blood virome of 10,585 individuals from the ChinaMAP

**DOI:** 10.1038/s41421-022-00476-1

**Published:** 2022-10-18

**Authors:** Jia Guo, Xuanlin Huang, Chenxi Zhang, Peide Huang, Yinhu Li, Fang Wen, Xiaoji Wang, Nanshan Yang, Min Xu, Yufang Bi, Guang Ning, Lin Li, Weiqing Wang, Yanan Cao

**Affiliations:** 1 SJTU-BGI Innovation Research Center, Shanghai, China; 2 BGI-ShenZhen, Shenzhen, Guangdong China; 3 National Center for Translational Medicine (Shanghai), Institute of Translational Medicine, Shanghai Jiao Tong University, Shanghai, China; 4 Department of Endocrine and Metabolic Diseases, Shanghai Institute of Endocrine and Metabolic Diseases, National Clinical Research Centre for Metabolic Diseases, State Key Laboratory of Medical Genomics, Key Laboratory for Endocrine and Metabolic Diseases of the National Health Commission, Research Unit of Clinical and Basic Research on Metabolic Diseases of Chinese Academy of Medical Sciences, Ruijin Hospital, Shanghai Jiao Tong University School of Medicine, Shanghai, China

**Keywords:** Genomic analysis, Bioinformatics

Dear Editor,

Understanding the composition of human blood virome is essential for safe blood transfusions and infectious disease surveillance and control. Screening the natural populations using sequencing technologies for the detection of known and novel viral pathogens could provide critical information for epidemiology and prevention of viral infections, vaccine development, and virus genomic investigations^1^. In addition, numerous common cancers are associated with oncogenic viruses, including Epstein-Barr virus (EBV), hepatitis B and C viruses (HBV and HCV), and human papillomavirus (HPV)^2^. The geographical and genetic diversity of population virome could be exploited for public health screening and prevention. Therefore, we analyzed the nonhuman sequencing reads in the 40× whole-genome sequencing (WGS) dataset of 10,585 individuals from the ChinaMAP^3^ to investigate the blood virome in the Chinese population.

Overall, we identified 14 viruses with a viral genome coverage of > 10% in the unmapped sequencing reads from the ChinaMAP dataset (Supplementary Fig. S1 and Table S1). The most prevalent anellovirus, including *Torque teno virus* (TTV) and TTV-like mini virus (TLMV), was identified in 76.7% of individuals. The prevalence of other 13 viruses with corresponding read abundance and coverage was shown (Fig. 1a). The human gammaherpes 4 (EBV) was detected in 30.3% of Chinese individuals, which was more than twice prevalence in a European dominant cohort (14%)^4^. The member of the betaherpesvirus family human herpesvirus 7 (HHV7) was detected in 13.2% of individuals, and the prevalence of HHV6A, HHV6B, and HHV5 (HCMV) was 0.36%, 1.09%, and 1.03%, respectively. The human endogenous retrovirus K (HERV-K), human mastadenovirus C, and HBV were identified in 8.20%, 2.41%, and 1.69% of individuals, respectively. The HPV subtypes *Gammapapillomavirus* 1, *Betapapillomavirus* 1, and *Alphapapillomavirus* 4 were found in 50 individuals. Notably, the oncogenic human polyomavirus 5, which was discovered in the Merkel cell carcinoma^5^, was detected in 29 individuals (0.27%). The DNA sequencing reads of influenza A virus was detected in 93 individuals, which may result from the injection of recombinant influenza vaccine or the reverse transcription by endogenous retroviral components^6^. Moreover, nonhuman origin viruses were also identified after strict filtering of false positives, which were not considered as infections due to interference from the environment and laboratory components (Supplementary Fig. S2).

**Fig. 1:**
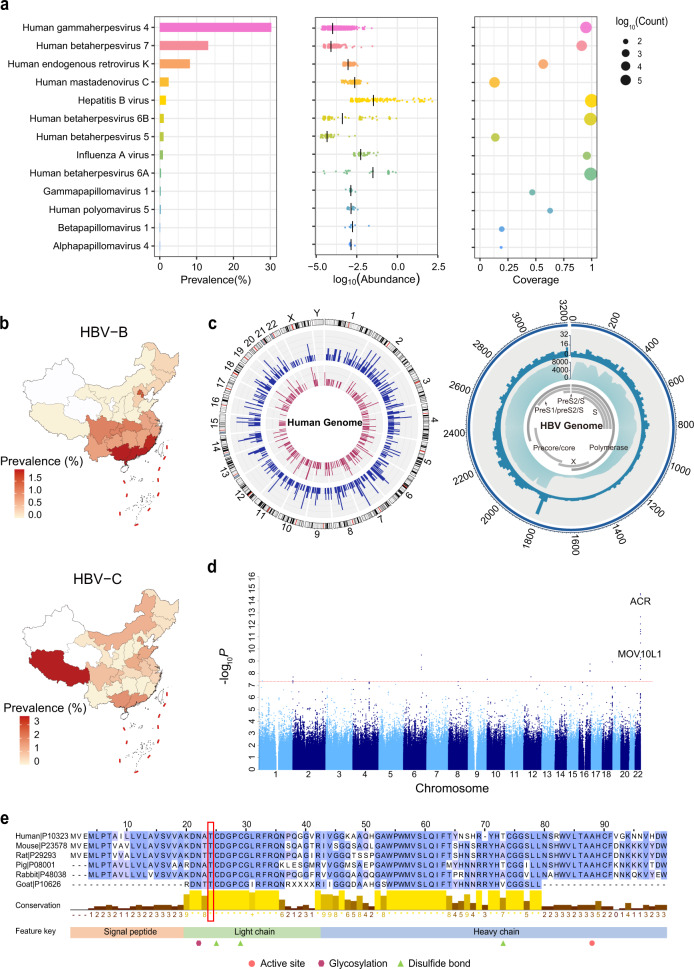
Landscape of blood virome in the Chinese population and analysis of HBV and HHV6. **a** The prevalence, abundance, and genome coverage of 13 viruses in human blood. **b** Geographic distribution of HBV-B and HBV-C in China. **c** The integration sites of HBV in the human (left) and HBV genomes (right). Left: red bars represent the integration sites of HBV-B, and blue bars represent the integration sites of HBV-C. Right: the dark blue histogram represents the integration sites in HBV genome, and the light blue histogram represents the viral genome coverage. The locations of the genes encoding HBV proteins are shown. The genomic positions of HBV genome are numbered. **d** Manhattan plot of GWAS for the HHV6 infection. **e** Conservation analysis of acrosin domains. The red box points the position of Thr24. The histograms represent the conservation scores calculated by Jalview (*full conservation; +full property conservation).

The virus read number per individual from various provinces showed no differences (Supplementary Fig. S3). We analyzed the viral abundance of top six prevalent viruses in the infected individuals and found more prevalent HHV7 and EBV in the younger (age ≤ 40) and older (age > 60) populations without gender differences. The geographic distribution and corresponding infected virus number and percentage were also analyzed (Supplementary Table S2). Moreover, we detected 179 phages that belong to 4 orders, 12 families and 88 genera in the blood virome in addition to the virus species, including the normal gut virus CrAssphage and numerous *Staphylococcus* phages (Supplementary Table S3).

The HBV infection mainly constituted HBV-B (*n* = 79) and HBV-C (*n* = 59) subtypes in 179 individuals. The HBV-B was more prevalent in southern China (Guangdong, Guangxi, Fujian provinces), whereas the HBV-C was prominently distributed in Tibet (Fig. 1b; Supplementary Fig. S4a). We analyzed the viral integration events in the human genome and found positive integration of HBV-B in 10 individuals and HBV-C in 18 individuals. The integration events presented a significant association with higher viral abundance (Supplementary Fig. S4b). The distribution of the detected integration breakpoints in the human and HBV genomes was analyzed (Fig. 1c). The HBV integration sites were randomly distributed without significant integration hotspots in the human genome. However, the integration breakpoints in the HBV genome showed a peak at the end of the HBV gp3 gene and start of the core gene, as reported previously^7^. Additionally, the phylogenetic tree of the HBV sequences from 56 individuals was constructed (variant number per sample ≥ 10 and allele frequency ≥ 10%) with corresponding geographic origins and 20 reference strains of HBV (Supplementary Fig. S4c). Two distinct clusters of HBV-B and HBV-C were observed, which was consistent with the genotypes of 20 reference strains. We also identified the HBV mutations that were critical for HBV-related hepatocarcinogenesis, including the basal core promoter (BCP) mutation T1762/A1764 and the precore region A1896 mutation. The mutation rate of BCP was higher in HBV-C (15.3%) than in HBV-B (2.5%), which was consistent with the previous reports^8^.

The human herpesviruses HHV4 (EBV), HHV5, HHV6A/B, and HHV7 were identified in our cohort. The geographic distribution of HHV6A/B indicated the relatively high prevalence in Hebei, Shaanxi, Shanxi, and Henan provinces (Supplementary Fig. S5a). Furthermore, we performed the genome-wide association study (GWAS) to explore the genetic susceptibility variants for virus infection (Supplementary Table S4). Strikingly, a missense variant (rs79314756) in the *ACR* gene (c. 71C>T, p. Thr24Met) was significantly associated with the HHV6 infection (*P* = 3.21 × 10^–15^) (Fig. 1d). The correlated significant variants locate in the *SHANK3* and *RABL2B* gene regions close to the lead SNP of *ACR* (Supplementary Fig. S5b–d). A previous study^9^ using low-pass sequencing data (~0.1×) of 141,431 Chinese women reported strong association between chromosomal integration of HHV6 in the infected population and the *MLC1-MOV10L1* locus close to the *ACR* gene region at 22q13.33. The *ACR* encodes the acrosin, which is a major acrosomal serine proteinase in the mature spermatozoa facilitating sperm penetration of the zona pellucida^10^. The missense mutation located in the highly conserved region next to the disulfide bond site (C25) and glycosylation site (N22) within the light chain of acrosin, suggesting that it might be functional for acrosin (Fig. 1e). We employed the AlphaFold2^11^ to predict the potential impact of *ACR* rs79314756 on the structure of acrosin, and the results indicated that it might affect the formation of polar interactions, including potential gain of polar contact between M24 and D26 (2.8 Å) and loss of contact between C25 and G27 (3.1 Å) (Supplementary Fig. S5e). The disulfide bond formation between amino acids 25 and 154 was predicted not to be disrupted. The HHV6A/B could selectively bind to the sperm head, which has an intact acrosome, and be transmitted to the uterus via sexual contact^12^. The most prevalent herpes type HHV6A/B was present in 13.5% of the sperm from 198 Danish donors^12^. We speculate that the *ACR* rs79314756 variant might enhance the activity of acrosin, thus facilitating the cleavage of C3 protein and promoting HHV6 virus transmission via the binding of membrane cofactor protein and oocyte receptors^13^.

Collectively, we illustrate the landscape of the human blood virome in a large cohort of Chinese population utilizing high-depth WGS data. The prevalence, viral abundance, and geographic distribution of 14 viruses were analyzed. Our results showed that the EBV was the most prevalent pathogenic virus present in 30% of individuals, and the integration events of HBV were correlated with the viral abundance in blood. A novel variant in *ACR* significantly associated with HHV6 infection was demonstrated, which could provide insights into understanding the susceptibility and pathogenesis of HHV6 transmission. These findings could provide essential information for the prevention of infectious diseases and the safety of blood transfusion in public health.

## Supplementary information

Supplementary information

Supplementary Tables

## Data Availability

The ChinaMAP variants database files can be downloaded from the ChinaMAP browser (www.mBiobank.com) following the regulation of the Human Genetic Resources Administration of China (HGRAC). Further analysis of sequencing data will be made available for collaborating researchers on request, dependent upon the approval of HGRAC.
