# Supplementary material for: The blood virome of 10,585 individuals from the ChinaMAP
Source: Cell Discov. 2022 Oct 18;8:113. doi: 10.1038/s41421-022-00476-1 (PMC9576698; doi:10.1038/s41421-022-00476-1)
Supplement: Supplementary file 1 — Supplementary information [file 41421_2022_476_MOESM1_ESM.pdf]

Supplementary Materials

Supplementary Methods

Data preparation and identification of unmapped sequences

All the sequencing data from the ChinaMAP database Phase 1 was analyzed (Supplementary, Fig. S2). We extracted read pairs with at least one of the reads not mapped to hg38 using SAMtools[1] v.1.9. Read pairs with more than 20% of bases and with a base quality below 20 were removed using fastp[2] v.0.19.5. Low complexity read pairs were identified and removed using PRINSEQ[3] v.0.20.4.

Identification of viral sequences

To identify viral sequences more accurately, we constructed a database including sequences from humans, Archaea (603 sequences), Bacteria (50062 sequences), and known vectors (3137 sequences) and a customized viral database composed of NCBI RefSeq, DDBJ and EMBL viral sequences (25644 viral sequences, date of download: 20201130). First, high-quality unmapped reads were aligned to our database using Kraken2[4] v.2.1.1, and then viral reads were identified and extracted according to the taxonomy id. Candidate reads were searched against our customized viral database by BLAST[5] v.2.7.1. Reads were then annotated at the species level. Viral hits were counted only if they met two requirements as follows: reads were aligned to only one species, reads had an e-value $< 1e\text{-}5$ and alignment length ≥ 80 bp. Three samples were filtered due to failure of quality control. We further filtered the reads that mapped to the same viral genome positions with very high frequency and the coverage distribution does not fit a Poisson distribution[6], as these reads could possibly be due to the contamination of

laboratory components[7]. Viral abundance was estimated by the following equation[8].

$$virus\ abundance = \frac{2 \times \frac{number\ of\ reads\ mapped\ to\ viral\ genome}{virus\ genome\ size}}{\frac{number\ of\ reads\ mapped\ to\ human\ genome}{human\ genome\ size}}$$

Subtype analysis and clusters of HBVs

HBV subtypes were acquired when annotations were executed at the subtype level. Reads from HBV subtype B and subtype C were aligned to Japan AB540582.1[9], taking into account the genetic information of Asian populations. Twenty strains (AB981581.1, AB981582.1, AB981583.1[10], AB602818.1, LC036263.1[11], LC057377.1, LC057378.1, LC060789.1, LC060790.1[12], AB176642.1[13], AB299858.1[14], AB540583.1, AB540584.1, AB540585.1[9], AB554017.1, AB554014.1, AB554015.1, AB554018.1, AB554019.1, AB554020.1)[15] from genotype B and genotype C each were also aligned to AB540582.1. We then constructed a differential position matrix and performed hierarchical clustering. The hierarchical clusters were converted into a tree[16] for visualization via a dendrogram[17].

Detection of virus integration events

After identification of viral reads by BLAST aligner, the paired-end reads that matched a viral species with only one mate were used to detect potential events of integration between the viral genome and the human genome. We investigated the BWA-alignment results of these unmatched mate reads. Both the perfect alignments to human genome and the split alignments where one part mapped to the human genome and the other part mapped to the same virus genome with the BLAST aligner were considered to be integration events.

Acrosin protein structure prediction

Conservation analysis of six different acrosins (P10323, P23578, P29293, P08001, P48038 and P10626), whose sequences were obtained from UniProt, was performed using Muscle[18] and Jalview[19]. AlphaFold2[20] was used to predict the protein structure of the wild-type and missense mutant (T24M) acrosin using the human acrosin amino acid sequence. The predicted structures with the highest confidence in PDB format were selected as PyMOL2 (The PyMOL Molecular Graphics System, Version 2.0 Schrödinger, LLC) input. Next, we used PyMOL2 to select all residues within 3.5 angstroms of ACR missense variant and then analyzed polar contacts changes. Ten repeated protein structure predictions (PDB files) of the wild-type and T24M mutation acrosin sequences were generated.

GWAS

To identify host genetic polymorphisms associated with viral infections, we performed a GWAS based on 9845 unrelated individuals without family relationship[21] and compared infected individuals to uninfected individuals in our study. Quality control procedures of SNPs included the following: 1) having a median depth greater than 8; 2) being within a low-complexity region (less than 7 single base repeat units); 3) having genotyping rate ≥ 90%; and 4) presenting HWE > 0.000001. EPACTS was employed to detect the associated signal with the top two principal components, age and gender, as covariates. The significantly associated loci were determined using a P value threshold $< 5 \times 10^{-8}$.

Statistical analysis

T-test was used to analyze the abundance difference between integrated and non-integrated HBV-infected samples. Statistical analysis of top 6 viruses' abundance across age and gender were also estimated using t-test. The difference in the abundance of geographical distribution was performed by ANOVA analysis.

References

1. Li, H., *et al.* The sequence alignment/map format and samtools. *Bioinformatics* **25**, 2078–2079 (2009).

2. Chen, S., Zhou, Y., Chen, Y. & Gu, J. Fastp: An ultra-fast all-in-one fastq preprocessor. *Bioinformatics* **34**, i884–i890 (2018).

3. Schmieder, R., & Edwards, R. Quality control and preprocessing of metagenomic datasets. *Bioinformatics* **27**, 863–864 (2011).

4. Wood, D.E., Lu, J. & Langmead, B. Improved metagenomic analysis with kraken 2. *Genome Biol.* **20**, 257 (2019).

5. Altschul, S.F., *et al.* Basic local alignment search tool. *J Mol Biol.* **215**, 403–410 (1990).

6. Shakir, K. DNA coverage prediction using aggregated poisson approximation. *Master's thesis, Harvard Extension School*. (2018).

7. Asplund, M., *et al*. Contaminating viral sequences in high-throughput sequencing viromics: a linkage study of 700 sequencing libraries. *Clin Microbiol Infect*. **25**, 1277–1285 (2019).

8. Moustafa, A., *et al.* The blood DNA virome in 8,000 humans. *PLOS Pathogens.* **13**, e1006292 (2017).

9. Mulyanto, *et al.* Identification and characterization of novel hepatitis b virus subgenotype c10 in nusa tenggara, indonesia. *Arch Virol.* **155**, 705–715 (2010).

10. Kamitsukasa, H., *et al.* Spontaneous reactivation of hepatitis b virus (hbv) infection in patients with resolved or occult hbv infection. *J Med Virol.* **87**, 589–600 (2015).

11. Inoue, J., *et al.* Shifting hepatitis b virus genotypes of acute hepatitis b patients in northeast japan. *J Med Virol.* **88**, 69–78 (2016).

12. Matsuo, J., *et al.* Clustering infection of hepatitis b virus genotype b4 among residents in vietnam, and its genomic characters both intra- and extra-family. *PLOS one.* **12**, e0177248 (2017).

13. Arbuthnot, P. & Kew, M. Hepatitis b virus and hepatocellular carcinoma. *Int J Exp Pathol.* **82**, 77–100 (2001).

14. Kato, H., *et al.* A fatal case of acute hepatitis b developed in a toluene abuser. *Clin J Gastroenterol.* **1**, 64–68 (2008).

15. Mulyanto, Depamede SN, Wahyono A *et al.* Analysis of the full-length genomes of novel hepatitis b virus subgenotypes c11 and c12 in papua, indonesia. *J Med Virol.* **83**, 54–64 (2011).

16. Paradis E, Schliep K. Ape 5.0: An environment for modern phylogenetics and evolutionary analyses in r. *Bioinformatics.* **35**, 526–528 (2019).

17. Yu, G. Using ggtree to visualize data on tree-like structures. *Curr. Protoc. Bioinformatics* **69**, e96 (2020).

18. Edgar, R.C. Muscle: A multiple sequence alignment method with reduced time and space complexity. *BMC Bioinformatics* **5**, 113 (2004).

19. Waterhouse, A.M., et al. Jalview version 2 – a multiple sequence alignment editor and

analysis workbench. *Bioinformatics* **25**, 1189–1191 (2009).

20. Jumper, J., *et al.* Highly accurate protein structure prediction with alphafold. *Nature* **596**, 583–589 (2021).

21. Cao, Y., et al. The ChinaMAP analytics of deep whole genome sequences in 10,588 individuals. *Cell Re*s. **30**, 717–731 (2020).

Supplementary Figures and legends

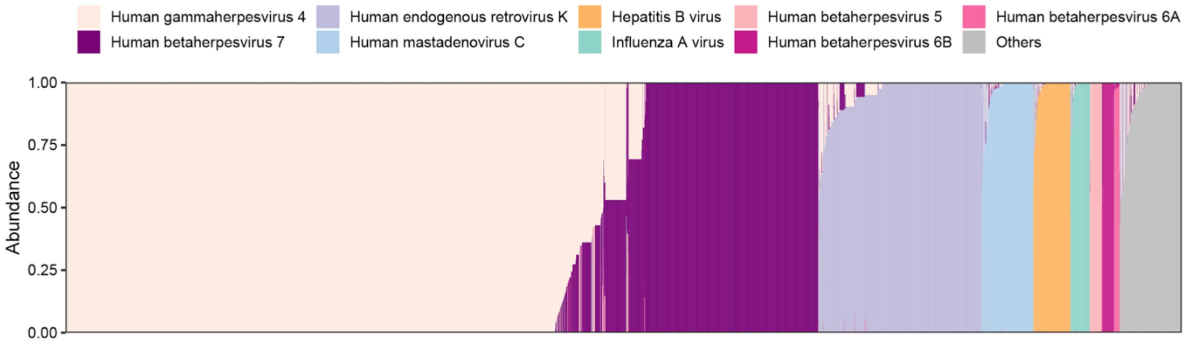

Fig. S1 The distribution of viral reads abundance in blood.

a

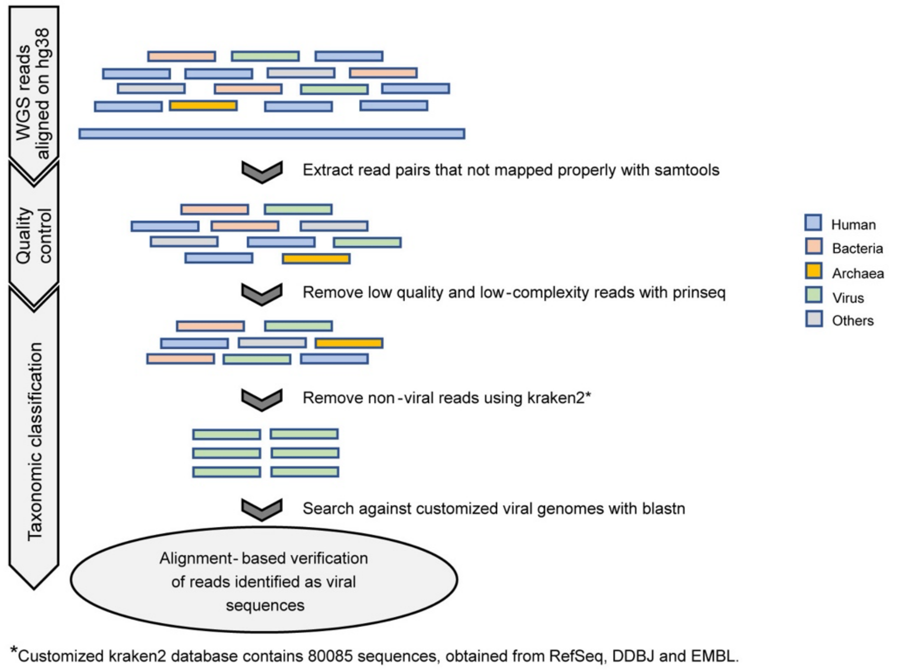

b

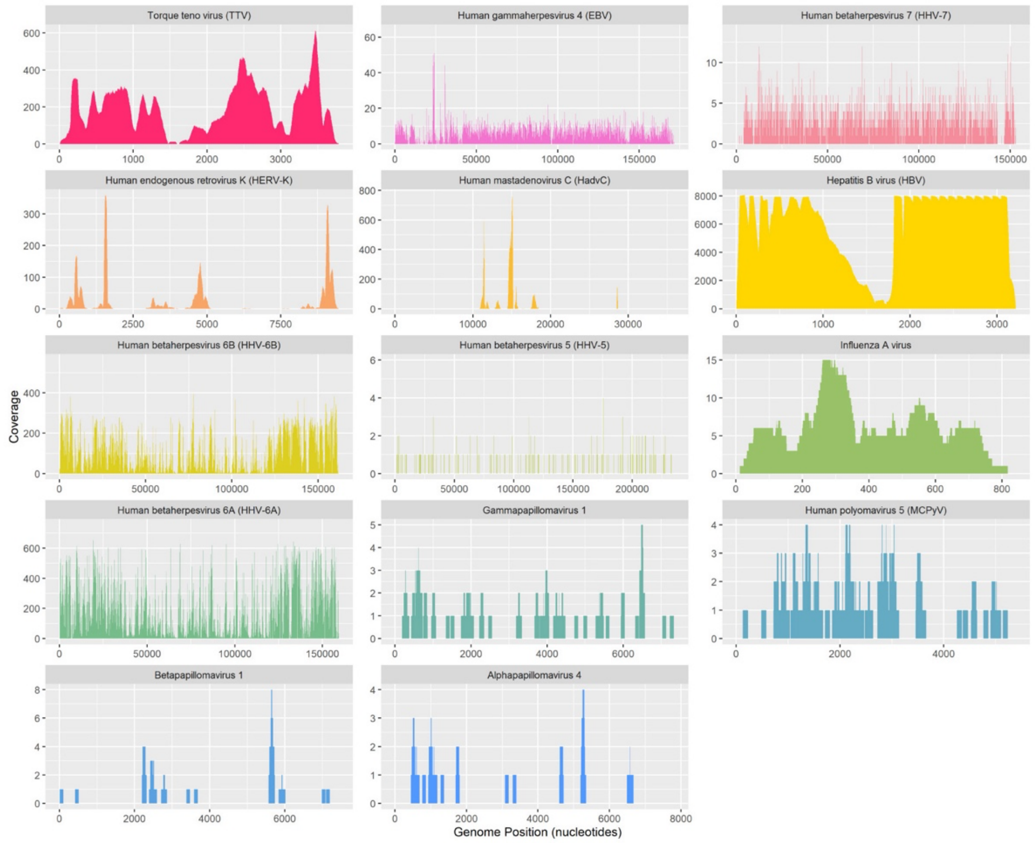

Fig. S2 Data processing and quality control. a. Pipeline of viral read identification from unmapped reads in our study. **b**. Genome coverage of viral sequence reads.

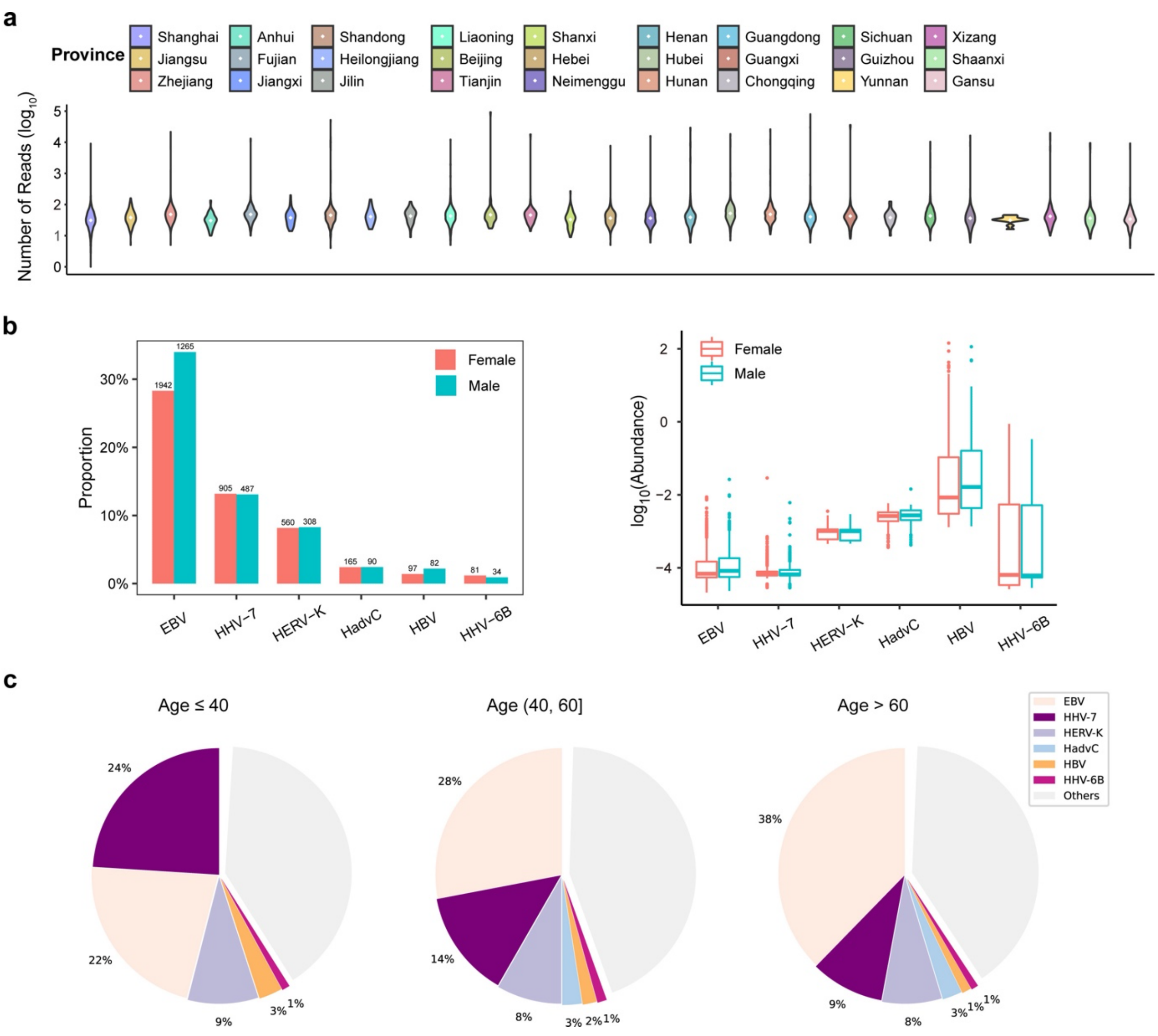

Fig. S3 Characteristics of viral carriers. a Viral sequence reads in individuals from different provinces of China. **b** The proportion and abundance of the top 6 predominant viruses in male and female carriers. **c** Percentage of the infections of viruses in different age groups.

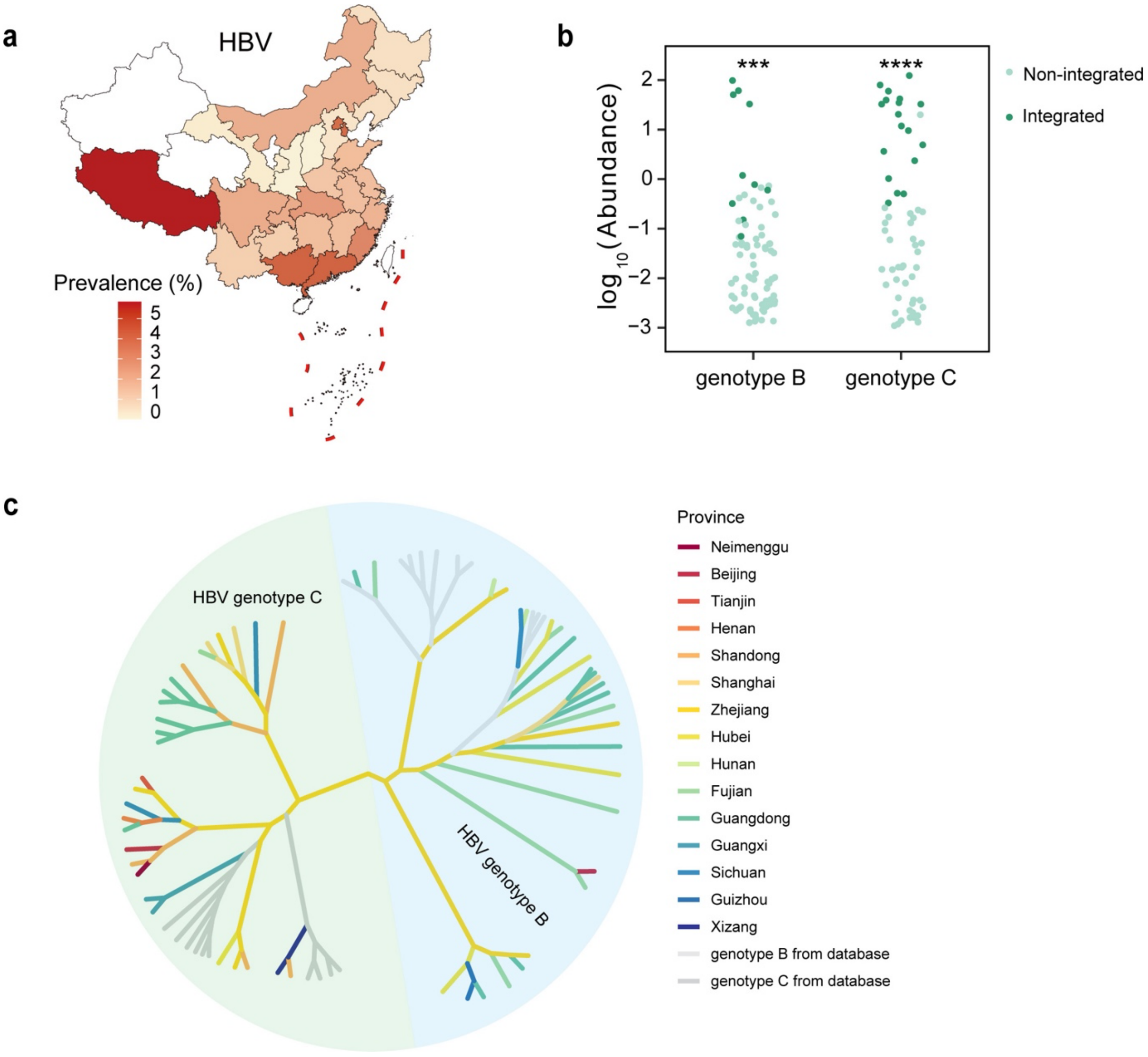

Fig. S4 Geographic distribution of HBV and analysis of integration events and phylogenetic tree of mutations. **a** Geographic distribution of HBV in China. **b** Read abundance of integrated and unintegrated HBV-B and HBV-C. The dark green color represents the integrated HBV carriers, and the light green color represents the unintegrated HBV carriers. Student's t test. ***$P < 0.001$, ****$P < 0.0001$. **c** Phylogenetic tree of HBV mutations.

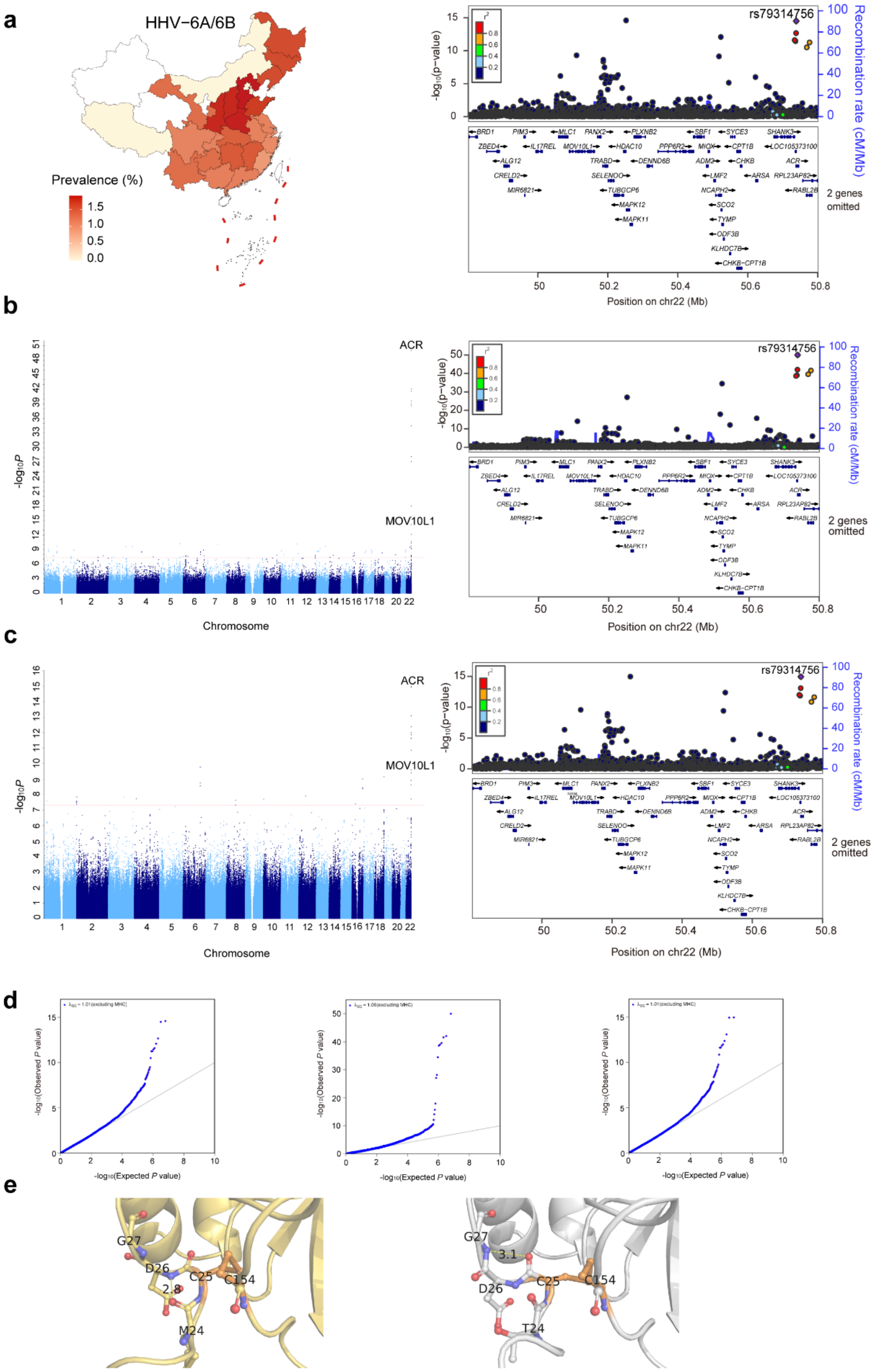

a
HHV−6A/6B
Prevalence (%)
1.5
1.0
0.5
0.0
rs79314756
-log10(p-value)
Recombination rate (cM/Mb)
2 genes omitted
Position on chr22 (Mb)
b
ACR
MOV10L1
-log10P
Chromosome
c
d
-log10(Observed P value)
-log10(Expected P value)
e
G27
D26
C25
C154
M24
2.8
3.1
T24

Fig. S5 Geographic distribution of HHV6A/6B and GWAS analysis. a Geographic distribution of HHV6A/6B (left); The *ACR* rs79314756 and the correlated SNPs (right). **b, c** Manhattan plot of independent GWAS of HHV6A (**b**) and HHV6B (**c**) infection. **d** QQ plot for the analyses of combined HHV6A/6B (left), HHV6A (middle) and HHV6B (right). **e** Predicted structures of mutant (24-Met, left) and wild-type (24-Thr, right) acrosin protein by Alphafold2.
